# Deep convolutional neural network for rib fracture recognition on chest radiographs

**DOI:** 10.3389/fmed.2023.1178798

**Published:** 2023-08-01

**Authors:** Shu-Tien Huang, Liong-Rung Liu, Hung-Wen Chiu, Ming-Yuan Huang, Ming-Feng Tsai

**Affiliations:** ^1^Department of Emergency Medicine, Mackay Memorial Hospital, Taipei, Taiwan; ^2^Department of Medicine, Mackay Medical College, New Taipei City, Taiwan; ^3^Graduate Institute of Biomedical Informatics, College of Medical Science and Technology, Taipei Medical University, Taipei, Taiwan; ^4^Big Data Research Center, College of Management, Taipei Medical University, Taipei, Taiwan; ^5^Division of Plastic Surgery, Department of Surgery, Mackay Memorial Hospital, Taipei, Taiwan

**Keywords:** deep convolutional neural network (DCNN), rib fracture, deep learning model (DLM), chest radiographs, artificial intelligence (AI), transfer learning

## Abstract

**Introduction:**

Rib fractures are a prevalent injury among trauma patients, and accurate and timely diagnosis is crucial to mitigate associated risks. Unfortunately, missed rib fractures are common, leading to heightened morbidity and mortality rates. While more sensitive imaging modalities exist, their practicality is limited due to cost and radiation exposure. Point of care ultrasound offers an alternative but has drawbacks in terms of procedural time and operator expertise. Therefore, this study aims to explore the potential of deep convolutional neural networks (DCNNs) in identifying rib fractures on chest radiographs.

**Methods:**

We assembled a comprehensive retrospective dataset of chest radiographs with formal image reports documenting rib fractures from a single medical center over the last five years. The DCNN models were trained using 2000 region-of-interest (ROI) slices for each category, which included fractured ribs, non-fractured ribs, and background regions. To optimize training of the deep learning models (DLMs), the images were segmented into pixel dimensions of 128 × 128.

**Results:**

The trained DCNN models demonstrated remarkable validation accuracies. Specifically, AlexNet achieved 92.6%, GoogLeNet achieved 92.2%, EfficientNetb3 achieved 92.3%, DenseNet201 achieved 92.4%, and MobileNetV2 achieved 91.2%.

**Discussion:**

By integrating DCNN models capable of rib fracture recognition into clinical decision support systems, the incidence of missed rib fracture diagnoses can be significantly reduced, resulting in tangible decreases in morbidity and mortality rates among trauma patients. This innovative approach holds the potential to revolutionize the diagnosis and treatment of chest trauma, ultimately leading to improved clinical outcomes for individuals affected by these injuries. The utilization of DCNNs in rib fracture detection on chest radiographs addresses the limitations of other imaging modalities, offering a promising and practical solution to improve patient care and management.

## 1. Introduction

Thoracic trauma accounts for 10% of trauma cases and 25–50% of deaths caused by trauma (1). Rib fractures are one of the most common manifestations of chest trauma and are associated with higher morbidity and mortality (2). Chest radiography is the most commonly used imaging tool for patients with chest trauma admitted to emergency rooms (3). However, previous studies have indicated that even with high specificity, chest radiographs do not show a high sensitivity in determining rib fractures (4), suggesting that half of the rib fractures cannot be detected by chest X-rays (5), and two-thirds of the rib fractures were solely observed through Computed tomography (CT) scans (6). A missing diagnosis of rib fracture has created tremendous stress on clinical emergency physicians facing chest trauma patients, and can lead to severe complications. While CT scans offer higher sensitivity, they are more costly and time-consuming. Point-of-care ultrasound can also be useful but requires additional time and is dependent on operator proficiency (4).

Human errors in healthcare can have serious consequences for patients. Diagnosing rib fractures is challenging for emergency physicians who treat patients with chest trauma, and misdiagnosis is not uncommon. In today’s overcrowded emergency rooms, the risk of errors is heightened, especially when it comes to diagnosing rib fractures from chest radiographs. Implementing Artificial Intelligence (AI) in the diagnosis and prognosis of diseases holds the potential to enhance healthcare quality (7, 8). In the case of chest trauma patients, the application of AI has the potential to reduce delays and transform workflows, ultimately revolutionizing the diagnosis and treatment of chest trauma and leading to improved outcomes.

While deep learning studies and applications for fracture identification on radiographs have emerged (9, 10), there remains a limited amount of research specifically focused on recognizing rib fractures in chest radiographs (11–13). Therefore, further research in the field is necessary.

In our study, we sought to address this issue by training deep convolutional neural network (DCNN) models on a comprehensive dataset of chest radiographs. The objective of our study was to gain novel insights and achieve outstanding results by developing highly accurate deep learning models for detecting rib fractures on plain chest radiographs. The main contribution of our study lies in addressing the challenge of diagnosing rib fractures from chest radiographs, which is a common yet often missed manifestation of chest trauma. a considerable number of rib fractures go undetected, leading to potential complications and increased stress on clinical emergency physicians. Our study yielded impressive validation accuracies for the trained models, with AlexNet achieving an outstanding accuracy of 92.6%, followed closely by GoogLeNet at 92.2%, EfficientNetb3 at 92.3%, DenseNet201 at 92.4%, and MobileNetV2 at 91.2%. These results demonstrate the potential of our deep learning models to assist doctors in accurately diagnosing rib fractures on plain chest radiographs. Implementing our trained models in clinical practice has the potential to significantly improve healthcare quality for chest trauma patients. By reducing delays, transforming workflows, and enhancing diagnostic accuracy, our approach can alleviate the workload of primary clinicians and enhance patient safety. The practical implementation of our trained deep learning models holds great promise for positively impacting patient care and outcomes in the field of chest trauma management.

## 2. Background

Before DCNN became available, conventional machine learning for fracture classification in medical images required image pre-processing and feature extraction (14, 15) before proceeding to the classification procedure. Edge detection had to be conducted first during image pre-processing, such as through a Harris corner detection (15), Gaussian edge detection, or Sobel edge detection (16). Further extraction of “useful features” that machine learning can learn from is the key step in conventional machine learning. Many algorithms are dedicated to feature extraction from images (17, 18). Unfortunately, none of the algorithms were fully applicable without human assistance. Although conventional machine learning used for fracture imaging determination has been considered acceptable in the past, the accuracy has been challenged owing to increased false positives. Kim et al. demonstrated the extraction and quantification of “useful features” for use in conventional machine learning in the task of rib fracture identification on plain radiographs (19); however false-positive cases were still frequently encountered.

The field of medical image detection and classification has witnessed a significant impact with the advancement of deep learning technology. One of the tremendous benefits of deep learning is that the machine automatically defines the features. Furthermore, deep learning has demonstrated its superiority over traditional machine learning approaches in image recognition, as indicated by several studies (20, 21). There is also an anticipated potential for deep learning in computer-aided diagnosis-based image analysis, including bone fracture identification (22).

Deep learning has proven to be highly effective in image identification (9, 23). However, building a practical convolutional neural network from scratch can be extremely challenging for most medical image researchers due to the requirement of vast amounts of data and substantial computing resources. As an alternative, transfer learning has gained popularity in recent years, whereby pre-trained convolutional neural networks (CNNs) that were initially trained for non-medical applications are utilized (24, 25). Similar techniques have been effectively applied in dermatological diseases (26), diabetic retinopathy (27), and lung diseases (28). Furthermore, the concept of using transfer learning from deep CNNs for bone fracture identification has been demonstrated in plain wrist radiographs (10). In this study, our goal was to employ transfer learning with pre-trained deep CNNs to detect rib fractures.

## 3. Materials and methods

The retrospective experiment undertaken was subjected to a comprehensive review and received approval from the Institutional Review Board of MacKay Memorial Hospital (MMH-IRB No. 20MMHIS483e), which included a thorough examination of all pertinent details. Moreover, all experiments carried out were conducted in strict compliance with relevant guidelines and regulations. Notably, the MacKay Memorial Hospital Institutional Review Board approved the waiver for informed consent.

### 3.1. Data acquisition

To gather the necessary data for our study, we collected a total of 5,000 chest X-ray images from patients who had radiological reports confirming the presence of at least one rib fracture. These images were obtained from the picture archiving and communication system of MMH (Medical Memorial Hospital) between the years 2015 and 2020.

To ensure the inclusiveness of our dataset, we incorporated both posterior–anterior (PA) view images and oblique view images of the chest radiographs. The selection criteria for identifying the appropriate images with rib fractures involved a thorough examination of the accompanying radiological reports. Only images that clearly indicated the presence of at least one rib fracture were included in our dataset. This rigorous selection process was essential to ensure the accuracy and reliability of our training data, allowing us to focus solely on rib fractures and avoid potential confounding factors.

By adhering to these stringent data collection procedures, we aimed to construct a robust and diverse dataset that encompassed a wide range of rib fracture cases, enabling our deep convolutional neural network models to learn and generalize from a comprehensive representation of rib fractures in patients with chest trauma.

### 3.2. Data pre-processing

**Figure 1:**
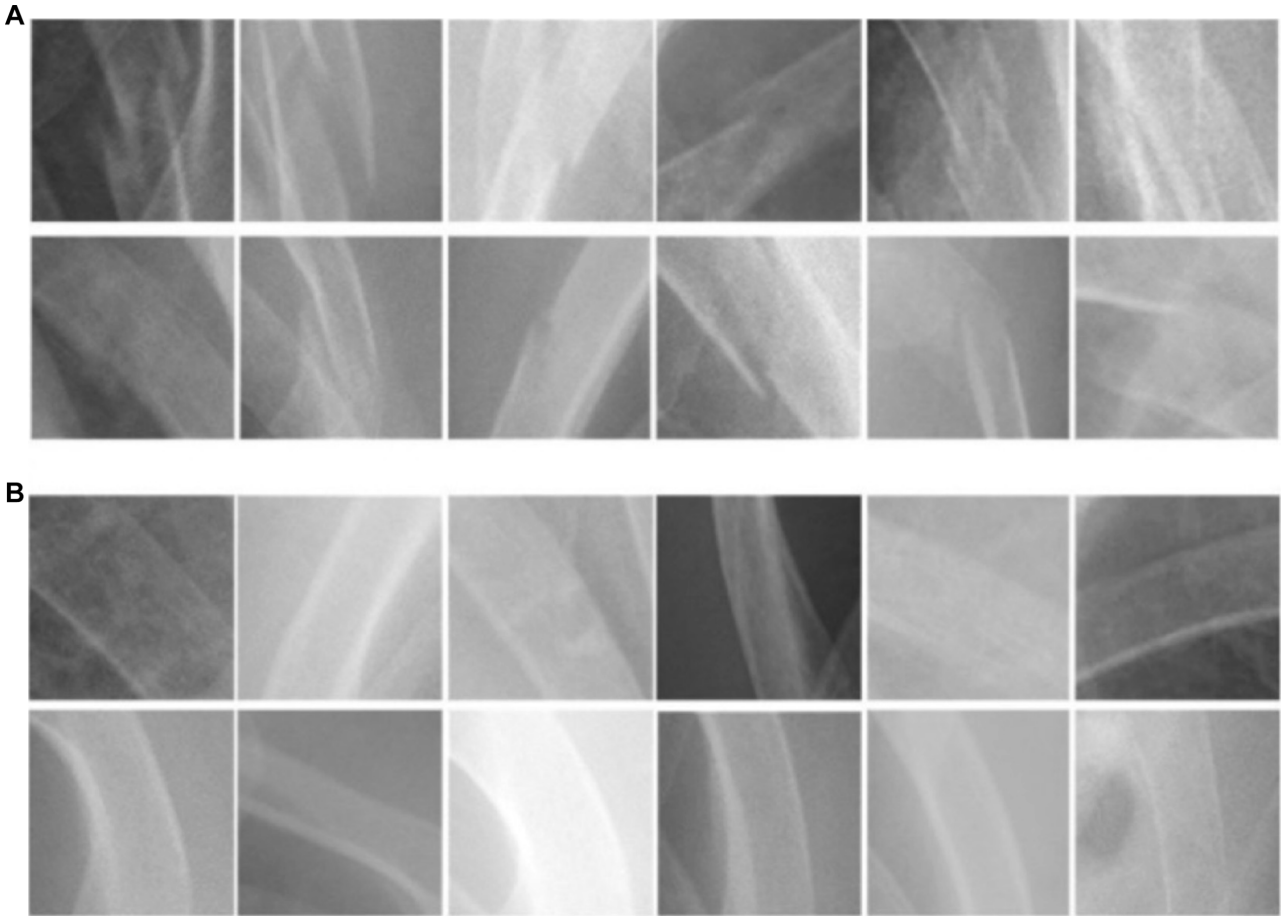
The morphological characteristics of **(A)** ribs with fractures and **(B)** ribs without fractures.

The images downloaded were transformed into PNG format and subjected to pre-processing to remove annotations and identities. Subsequently, a clinical emergency physician with 15 years of experience selected and gathered 2000 square-shaped patches as bonding boxes to delineate “fractured ribs” and “non-fractured ribs,” based on formal radiological reports. These patches were then designated as Regions of Interest (ROIs) and utilized for training our Convolutional Neural Network (CNN) model. Fractured rib bones on chest X-rays are identified by deformities in their shape, such as breaks in the bone cortex or fragments overriding each other (19, 29). Figure 1 demonstrates examples of various shapes of fractured and normal ribs. However, initial attempts of scanning the chest X-ray patches using a sliding window approach led to misidentification of background noise as positive signals for rib fractures. As a solution, we selected 2,000 Regions of Interest (ROIs) from the chest X-rays that contained non-rib-related information and categorized them as “background.” We also experimented with different pixel resolutions (80 × 80, 100 × 100, and 150 × 150) during the selection process and ultimately determined that 128 × 128 pixels were the most appropriate for analysis. This resolution was chosen to fully capture the lesion while avoiding excessive background noise.

### 3.3. Model training

In this study, the computer operating system utilized was Windows 10, developed by Microsoft Corporation, Redmond, WA, USA. The software used was MATLAB R2020b, developed by MathWorks, Inc., Natick, MA, USA, which included a built-in Neural Network Toolbox. The graphics processing unit (GPU) utilized was a GeForce GTX 1070, developed by Nvidia Corporation, Santa Clara, CA, USA.

To accomplish our objective, we employed five pre-trained deep convolutional neural networks (DCNNs) with open-source codes, namely AlexNet, GoogLeNet, EfficientNetb3, DenseNet201 and MobileNetV2 for transfer learning. AlexNet (30), previously trained for the large-scale ImageNet visual recognition competition (ILSVRC-2010, 2012), comprises five convolutional layers and three fully connected layers. On the other hand, GoogLeNet (31) was also trained for ILSVRC and emerged as the winner of the ILSVRC-2014. The architecture of GoogLeNet, referred to as an “inception module,” features 22 layers of a deep CNN with 12-fold fewer parameters than AlexNet. EfficientNetb3 (32) is an efficient and accurate CNN model for computer vision tasks. It utilizes compound scaling to optimize depth and width, achieving state-of-the-art performance. DenseNet201 (33) is characterized by its deep and densely connected neural network structure, facilitating efficient information flow and strong feature extraction. MobileNetV2 (34) is an efficient CNN architecture optimized for mobile and embedded devices. It utilizes depth wise separable convolutions and inverted residuals to achieve high performance while minimizing computational cost and model size. This makes it a powerful architecture for a range of computer vision tasks. Although those DCNNs were initially based on non-radiological images, we adapted them to construct the models for identifying rib fractures in our study.

In order to enhance the quantity and diversity of our dataset, we incorporated various data augmentation techniques during model training, including flipping, rotation, and parallel shifting. This approach served to improve the performance of our deep convolutional neural network (DCNN) while simultaneously mitigating the risk of overfitting (35). Following data augmentation, the enlarged dataset was partitioned into training (70%) and validation (30%) sets to facilitate the development and evaluation of our models. This process enabled us to train our DCNN on a sizeable and diverse dataset, while also ensuring that our models were able to generalize to new data beyond the training set. Regarding the training hyperparameters, we carefully selected and tuned them to optimize the training process. All five deep convolutional neural network (DCNN) models were trained using a fixed learning rate of 0.0001, with 50 epochs and a batch size of 32 images. This standardized training setup maintained consistency across the models and allowed for an adequate number of iterations to facilitate effective learning and optimization.

### 3.4. DCNN models validation statistics

Ultimately, we subjected our deep convolutional neural network (DCNN) models, trained to classify rib images into three categories (background, fractured, and non-fractured rib), to a rigorous evaluation process. Our assessments were comprehensive, encompassing validation accuracy, sensitivity, specificity, positive predictive value, negative predictive value, and F1 score, as well as the calculation of the area under the receiver operator characteristic (ROC) curve (AUC).

**Figure 2:**
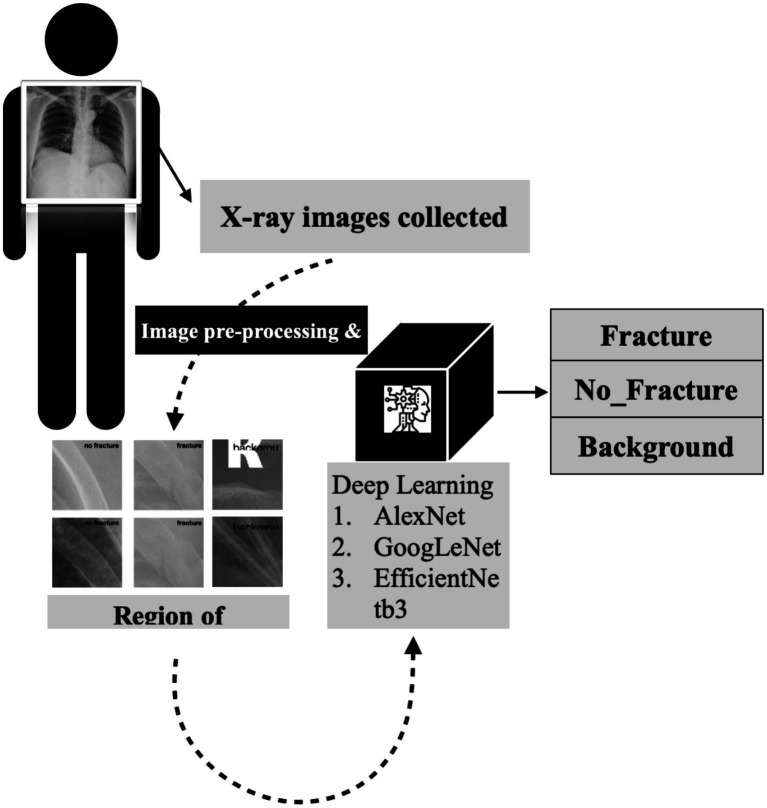
The detailed flowchart of our research methodology for developing a deep convolutional neural network for rib fracture recognition on chest radiographs.

A detailed visual representation of the study’s experimental design is presented in Figure 2, outlining the various stages of the research methodology and facilitating a clear understanding of our study’s approach and findings.

### 3.5. Visual explanation of the trained model

To enhance the interpretability of our models, we implemented the Gradient-weighted Class Activation Mapping (Grad-CAM) technique, developed by Selvaraju et al. (36). By leveraging the classification score gradient with respect to the convolutional features identified by the network, this innovative approach facilitates the interpretation of deep learning models. With Grad-CAM, we were able to generate a heatmap visualization that highlights the input regions that are most significant for accurate classification. In essence, this technique empowers convolutional neural network (CNN) models to be more transparent and interpretable, enabling researchers to better understand the underlying features and patterns that contribute to successful model performance.

## 4. Results

### 4.1. Comparative validation results of five deep learning models

**Figure 3:**
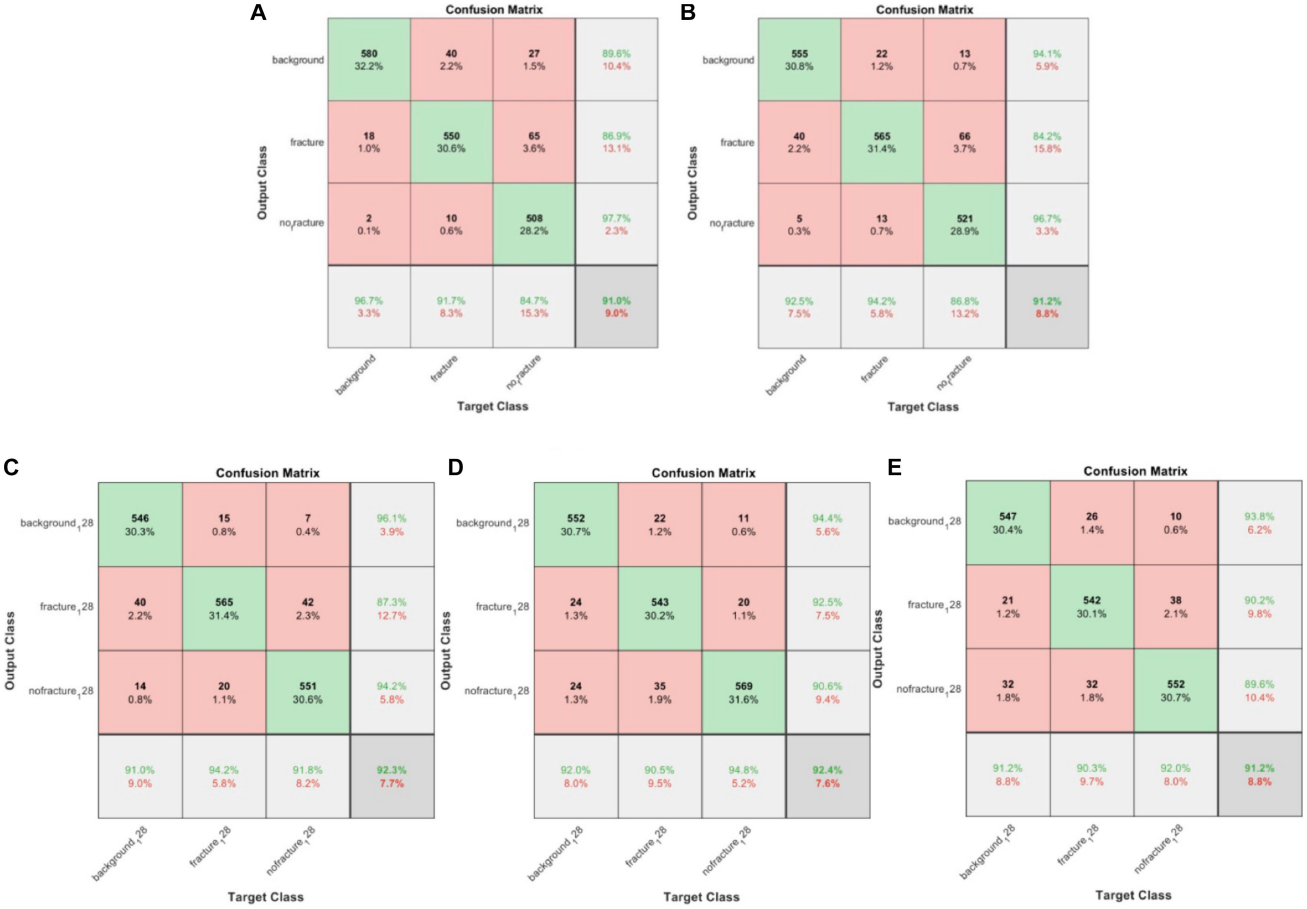
Confusion matrix for the three-class classification deep convolutional neural network (DCNN) models, including **(A)** AlexNet, **(B)** GoogLeNet, **(C)** EfficientNetb3, **(D)** DenseNet201, and **(E)** MobileNetV2.

**Figure 4:**
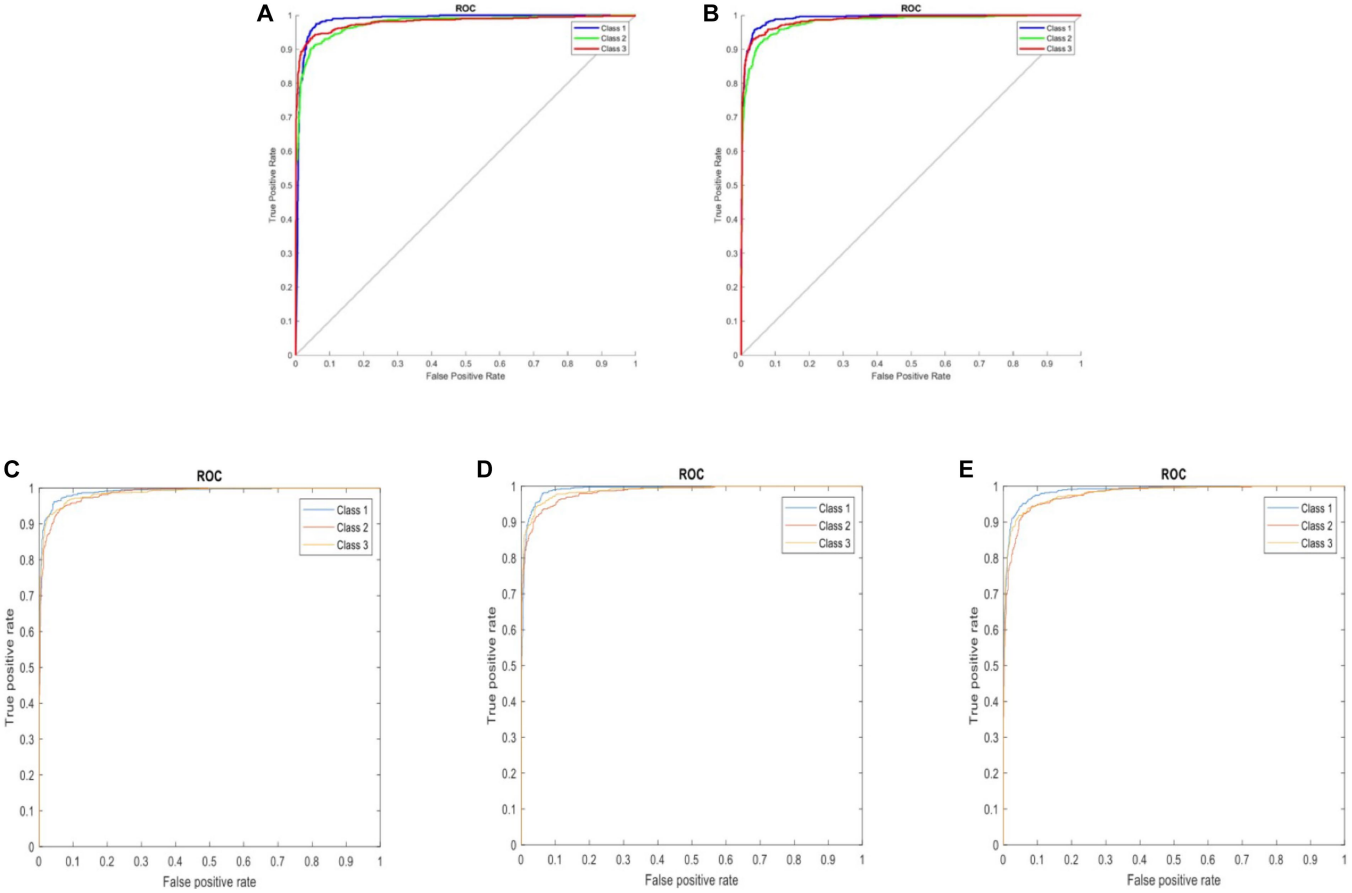
ROC curves comparing five three-class DCNN models: **(A)** AlexNet, **(B)** GoogLeNet, **(C)** EfficientNetb3, **(D)** DenseNet201, and **(E)** MobileNetV2. Classes represented: 1 – background, 2 – fractured rib, and 3 – non-fractured rib.

Following the completion of model training, all five distinct deep learning models underwent evaluation using the identical validation dataset, each exhibiting outstanding performance. The results of these models are summarized in Table 1. The AlexNet transfer learning model achieved an accuracy of 92.6%, with a sensitivity of 91.7%, specificity of 93.1%, positive predictive value (PPV) of 86.9%, negative predictive value (NPV) of 95.7%, and an F1 score of 0.890. Similarly, the GoogLeNet transfer learning model achieved an accuracy of 92.2%, with a sensitivity of 94.2%, specificity of 91.2%, PPV of 84.2%, NPV of 96.9%, and an F1 score of 0.889. The EfficientNetb3 transfer learning model achieved an accuracy of 92.3%, with a sensitivity of 94.2%, specificity of 92.3%, PPV of 87.3%, NPV of 97.0%, and an F1 score of 0.904. Furthermore, the DenseNet201 transfer learning model achieved an accuracy of 92.4%, with a sensitivity of 90.5%, specificity of 96.3%, PPV of 92.5%, NPV of 95.3%, and an F1 score of 0.915. Lastly, the MobileNetV2 transfer learning model achieved an accuracy of 91.2%, with a sensitivity of 90.3%, specificity of 95.1%, PPV of 90.2%, NPV of 95.2%, and an F1 score of 0.902. Confusion matrix results for each model are depicted in Figure 3, and the ROC curves, shown in Figure 4, indicate the AUC values of 0.976 for AlexNet, 0.979 for GoogLeNet, 0.984 for EfficientNetb3, DenseNet201, and 0.978 for MobileNetV2.

**Table 1:** The comparison among each deep learning model.

DCNN	Accuracy	Sensitivity	Specificity	PPV	NPV	F1 score	AUROC (95% CI)
AlexNet	0.926	0.917	0.931	0.869	0.957	0.890	0.976 (0.9762 ~ 0.9864)
GoogLeNet	0.922	0.942	0.912	0.842	0.969	0.889	0.979 (0.9736 ~ 0.9853)
EfficientNetb3	0.923	0.942	0.923	0.873	0.970	0.904	0.984 (0.9773 ~ 0.9865)
DenseNet201	0.924	0.905	0.963	0.925	0.953	0.915	0.984 (0.9749 ~ 0.9862)
MobileNetV2	0.912	0.903	0.951	0.902	0.952	0.902	0.978 (0.9718 ~ 0.9833)

### 4.2. Grad-CAM for the interpretation of the model

**Figure 5:**
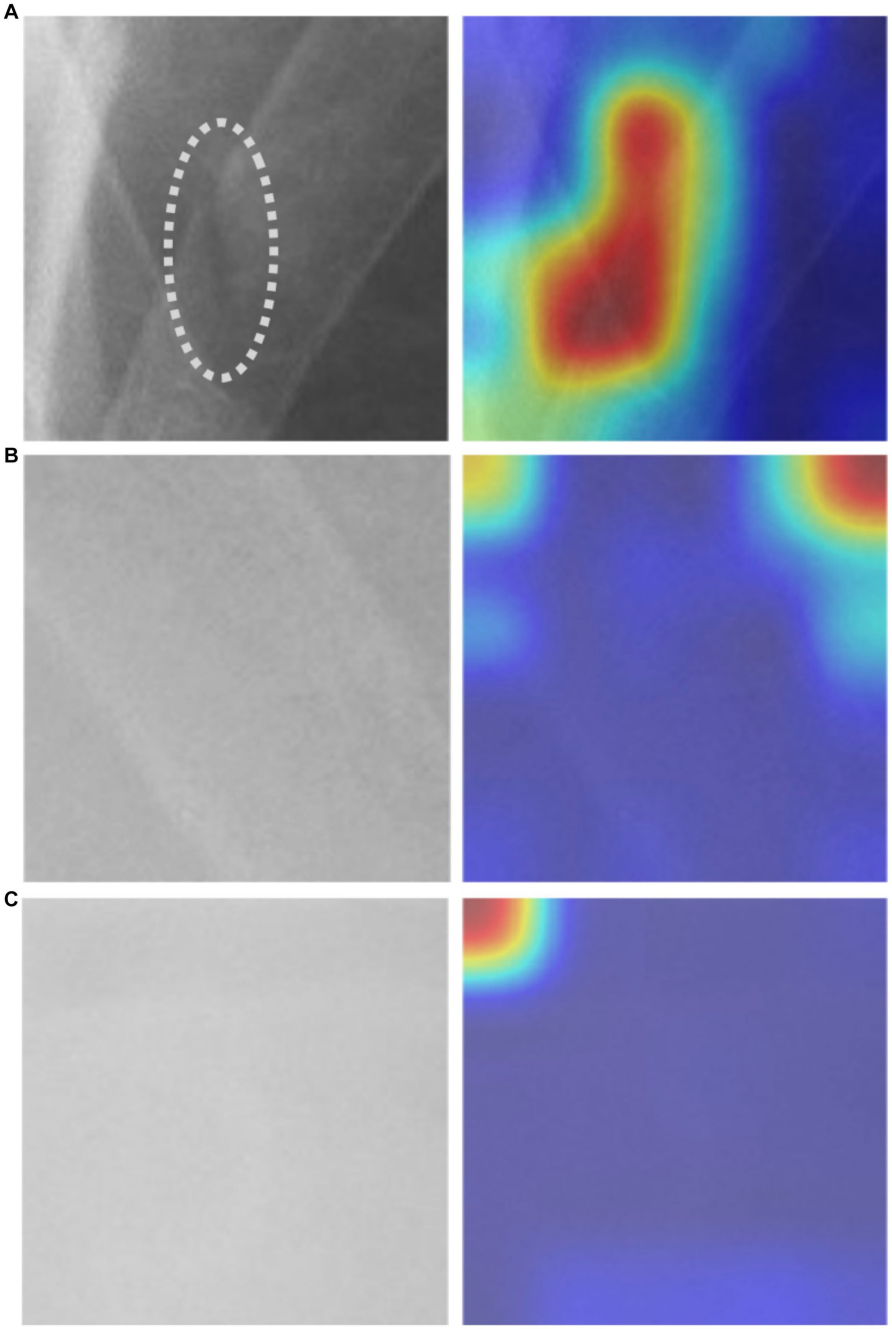
The Grad-CAM visualization: **(A)** an image depicting a fractured rib with a corresponding mark, alongside its respective Grad-CAM visualization, **(B)** non-fractured rib image with Grad-CAM, and **(C)** background image with Grad-CAM.

In our study, we utilized the Gradient-weighted Class Activation Map technique (Grad-CAM) to obtain a comprehensive visualization of the fracture recognition model that we trained. This technique enabled us to identify the key regions within the model that significantly influenced the classification decision. As illustrated in Figure 5, our model generated a consistent Grad-CAM pattern, highlighting the decision-making process of the classification network, shedding light on the factors contributing to the ultimate classification outcome. This visualization technique adds to the interpretability of our model and enhances its clinical utility.

## 5. Discussion

The advancement of developing a computer-based technology for detecting rib fractures in chest X-ray images has the potential to prevent misdiagnosis and improve the quality of healthcare for patients, thereby avoiding severe complications resulting from delayed or inappropriate treatment. However, this is a challenging task due to inherent limitations of chest radiographs, such as overlapping organs, bones such as the clavicle and spine, and other ribs that may impede accurate image interpretation (19).

For our study, we opted to manually select the region of interest (ROI) with a pixel resolution of 128 × 128 for the training of our deep convolutional neural network (CNN) models. Medical images can often contain lesions, such as fractures, as well as irrelevant noise. It is widely recognized that using an ROI can significantly improve the accuracy of both conventional machine learning (19) and deep learning methods (37). Nevertheless, ensuring the suitability of the selected ROI necessitates the involvement of experts possessing adequate knowledge to verify whether the ROI accurately encompasses the ground truth of the primary disease information (38).

Compared to conventional machine learning methods used for rib fracture detection on chest radiographs, the deep learning method offers several advantages. It eliminates the need for manual feature extraction and showcases superior capability. Our study’s five models performed exceptionally well, achieving impressive AUC values: 0.976 for AlexNet, 0.979 for GoogLeNet, 0.984 for EfficientNetb3 and DenseNet201, and 0.978 for MobileNetV2. By utilizing transfer learning techniques from a deep convolutional neural network (CNN) pre-trained on non-medical images, our models demonstrate the ability to accurately recognize rib fractures in plain chest radiographs.

Comparing to existing studies focusing on rib fracture recognition in chest radiographs using deep learning, one study employed You Only Look Once v3 (YOLOv3) and achieved an accuracy of 85.1% and an AUC of 0.92 (11). Another study specifically addressed the challenge of limited annotated positive samples by employing a mixed supervised learning technique, resulting in an accuracy of 87.8% (12). Additionally, in a study specifically targeting a young age cohort, ResNet-50 achieved an AUC-ROC of 0.74, while ResNet-18 achieved an AUC-ROC of 0.75 for identifying rib fractures on chest radiographs (13). However, when compared to our study, these approaches exhibited inferior performance. Moreover, we also compared our study with others focusing on deep learning models for rib fracture recognition in CT scans. One study achieved a validating F1-score of 0.888, with precision and recall values of 0.864 and 0.914, respectively (39). Another study showed higher detection efficiency for fresh and healing fractures compared to old fractures, with F1-scores of 0.849, 0.856, and 0.770, respectively (40). Our study’s accuracy is comparable to these results. Considering the expense and time required for CT imaging, deep learning on chest radiographs is a more practical approach for first-line clinical practitioners.

Although our study did not compare our model’s performance to that of doctors, previous literature suggests that the missed rib fractures rate could be as high as 20.7% (41). Given our model’s high accuracy, we believe it could be a useful tool in reducing missed diagnoses, especially since even minor missed injuries can have important consequences for patients, clinicians, and radiologists, as noted by Pinto et al. (42). In future applications, the utilization of the deep learning model holds promise in providing timely alerts to healthcare professionals regarding potential fractures observed in chest radiographs. This valuable tool would prompt primary care physicians to exercise heightened vigilance and consider conducting supplementary diagnostic procedures such as sonography or CT scans to confirm the presence of a fracture, thereby minimizing the risk of overlooking such injuries. By integrating this advanced technology into clinical practice, the deep learning model demonstrates its potential to significantly enhance fracture detection and ensure comprehensive patient care.

## 6. Limitations and future directions

Our study has demonstrated that the concept of transfer learning using pre-trained deep convolutional neural networks (DCNNs) can be successfully applied in recognizing rib fractures in chest radiographs. However, we acknowledge a limitation in our study: only one experienced clinician labeled a substantial number of ROIs without reference from another expert. This introduces the potential for bias when evaluating the impact of DCNN training. In the future, to address this issue when precise delineated labels are scarce, we may consider adopting the Mixed Supervised Learning method (12). This approach can help reduce the need for fine annotation tasks and further enhance the training process. Furthermore, the application of DCNN has often been criticized for being a “black box” with no clear explanations for its underlying processes. Nevertheless, as long as robust testing methods are employed to prove its acceptable level of safety and efficacy, this cutting-edge technology cannot be disregarded (10). Additionally, we utilized the technique of Grad-CAM to visualize the classification decision of the deep learning model, which further aided in providing an explanation for the results.

Regarding the representativeness of the dataset, we acknowledge that there may be inherent biases in the dataset, including potential variations in imaging protocols and patient demographics. It is important to consider these factors when interpreting the generalizability of our results to other healthcare settings. To further address this limitation, future studies should aim to include multicenter datasets with diverse patient populations to ensure the broader applicability of our findings.

**Figure 6:**
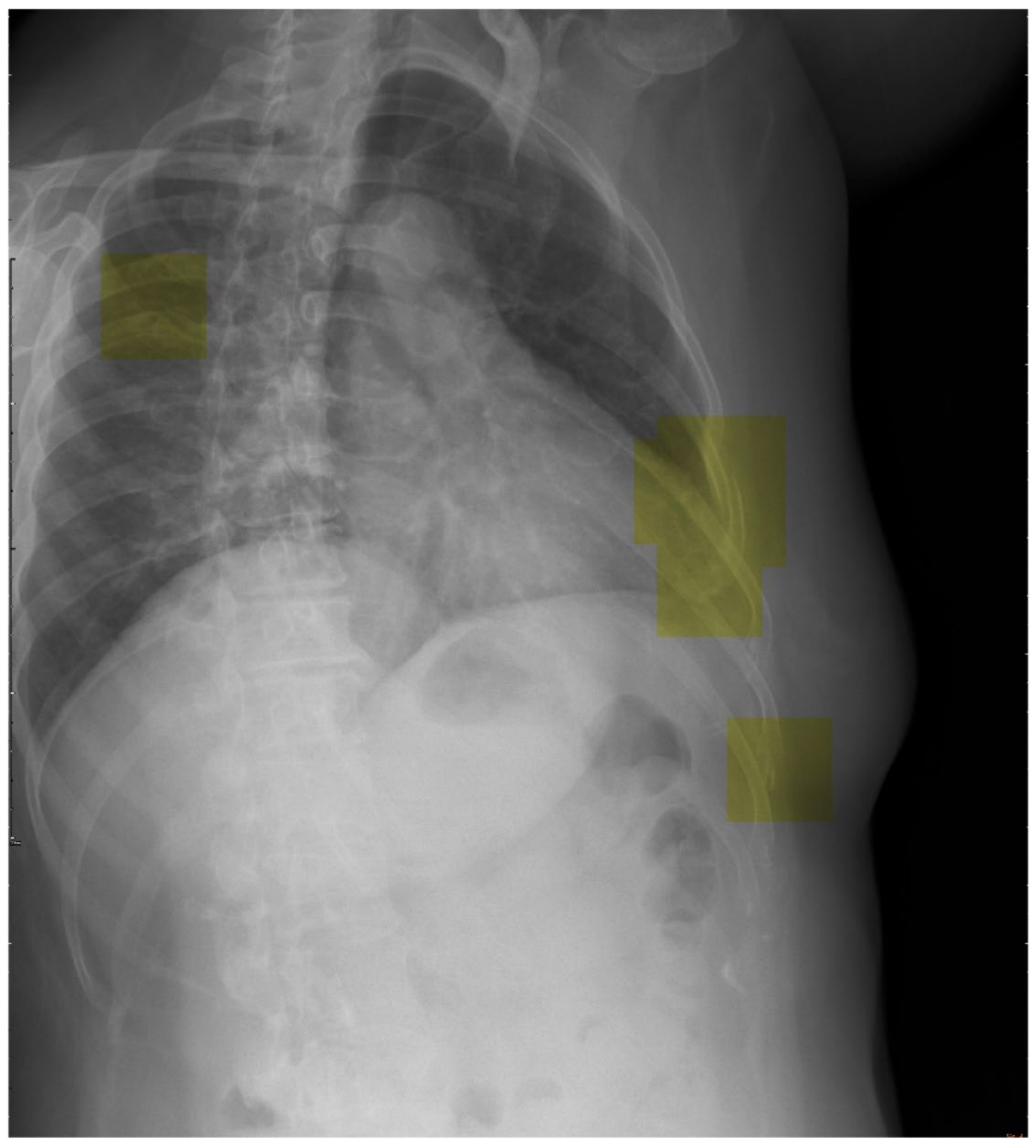
Visual representation showcasing the application of inference (highlighted by the yellow box) in our model for detecting potential rib fractures on chest radiographs.

To advance future research efforts, we should endeavor to implement the trained DCNN model utilizing a patch-based sliding window scanning method (43, 44) to interpret chest radiographs obtained from trauma patients in an emergency setting and highlight the suspected fractured ribs. Therefore, we have the opportunity to deploy this advanced tool as a computer-aided diagnostic system in the clinical setting, effectively preventing instances of misdiagnosis. Moreover, we provide a visual depiction (Figure 6) that highlights the utilization of inference (represented by the yellow box) in our model for the detection of potential rib fractures on chest radiographs.

In terms of the integration of AI-assisted diagnosis into clinical practice, future research should examine the impact of incorporating the trained DCNN models into the existing workflow of healthcare professionals. Assessing the acceptance, usability, and effectiveness of these models in real clinical settings will provide valuable insights into the practical implementation and potential benefits for both patients and medical practitioners.

## 7. Conclusion

In our study, we have convincingly demonstrated the proficient capabilities of transfer learning-based deep convolutional neural network (DCNN) models in accurately identifying fractured ribs in chest radiographs. By utilizing transfer learning with well-established architectures such as AlexNet, GoogLeNet, EfficientNetb3, DenseNet201, and MobileNetV2, we achieved validation accuracies ranging from 91.2 to 92.6%, highlighting the remarkable performance of these models. In comparison to existing studies focusing on rib fracture recognition in chest radiographs using deep learning, our study outperformed these approaches. Furthermore, when comparing our study with those focusing on deep learning models for rib fracture recognition in CT scans, our accuracy was found to be comparable.

The successful implementation of this methodology paves the way for the development of a computer-aided system that can effortlessly recognize suspected regions of rib fractures and promptly alert clinicians. Such advancements represent significant strides toward elevating the standard of care provided in high-stress emergency settings, where timely and decisive action is often critical.

Nevertheless, it is essential to acknowledge the limitations of our study. While our DCNN models demonstrated exceptional performance, there may still be cases where rib fractures are missed or misdiagnosed. Additionally, the generalizability of our findings to diverse patient populations and imaging settings should be carefully considered.

In terms of future works, several areas warrant further exploration. Firstly, refining the computer-aided system to enhance its performance and robustness in real-world clinical scenarios is crucial. This includes addressing potential challenges such as image variability, limited data availability, and integrating the system seamlessly into existing clinical workflows. Furthermore, conducting prospective studies and involving a larger patient cohort would strengthen the evidence base and validate the utility of the developed system in clinical practice. Moreover, incorporating clinical feedback and iterative model refinement can contribute to continuous improvement and adaptation of the system to evolving clinical needs. The cautious integration of this assistance system holds great potential in enhancing workflow productivity, reducing the likelihood of errors, and ultimately minimizing harm to patients.

## Data availability statement

The raw data supporting the conclusions of this article will be made available by the authors, without undue reservation.

## Ethics statement

The retrospective experiment undertaken was subjected to a comprehensive review and received approval from the Institutional Review Board of MacKay Memorial Hospital (MMH-IRB No. 20MMHIS483e), which included a thorough examination of all pertinent details. Moreover, all experiments carried out were conducted in strict compliance with relevant guidelines and regulations. Notably, the MacKay Memorial Hospital Institutional Review Board approved the waiver for informed consent.

## Author contributions

S-TH and M-YH conceived of the presented idea. S-TH and H-WC developed the methods. L-RL and M-FT provided the clinical insights. S-TH carried out the experiment, trained the models, wrote the manuscript. H-WC supervised the study. All authors discussed the results, reviewed the manuscript, and approved the final manuscript.

## Funding

This research was financially supported by Ministry of Science and Technology, Taiwan, R.O.C. under Grant No. and MOST 110-2221-E-038-006.

## Conflict of interest

The authors declare that the research was conducted in the absence of any commercial or financial relationships that could be construed as a potential conflict of interest.

## Publisher’s note

All claims expressed in this article are solely those of the authors and do not necessarily represent those of their affiliated organizations, or those of the publisher, the editors and the reviewers. Any product that may be evaluated in this article, or claim that may be made by its manufacturer, is not guaranteed or endorsed by the publisher.
